# Synthesis and Characterization of Polyethylene Glycol Mediated Silver Nanoparticles by the Green Method

**DOI:** 10.3390/ijms13066639

**Published:** 2012-05-30

**Authors:** Kamyar Shameli, Mansor Bin Ahmad, Seyed Davoud Jazayeri, Sajjad Sedaghat, Parvaneh Shabanzadeh, Hossein Jahangirian, Mahnaz Mahdavi, Yadollah Abdollahi

**Affiliations:** 1 Department of Chemistry, Faculty of Science, Universiti Putra Malaysia, 43400 UPM Serdang, Selangor, Malaysia; E-Mails: mansorahmad@gmail.com (M.B.A.); kamran.jahangirian@gmail.com (H.J.); mahnaz.chem@gmail.com (M.M.); 2 Materials and Energy Research Center, Meshkin-Dasht Road, Karaj 3177983634, Iran; 3 Institute of BioSciences, Universiti Putra Malaysia, 43400 Serdang, Selangor Darul Ehsan, Malaysia; E-Mail: davoudjazayeri@yahoo.com; 4 Department of Chemical Engineering, Faculty of Engineering, Islamic Azad University, Malard Branch 3169153174, Iran; E-Mails: sajjadsedaghat@yahoo.com (S.S.); parvaneh.shabanzade@gmail.com (P.S.); 5 Advance Materials and Nanotechnology Laboratory, Institute of Malaysia Advance technology, 43400 UPM Serdang, Selangor, Malaysia; E-Mail: yadollahabdollahi@yahoo.com

**Keywords:** silver nanoparticles, green chemistry, polyethylene glycol, transmission electron microscopy, reaction time effect

## Abstract

The roles of green chemistry in nanotechnology and nanoscience fields are very significant in the synthesis of diverse nanomaterials. Herein, we report a green chemistry method for synthesized colloidal silver nanoparticles (Ag NPs) in polymeric media. The colloidal Ag NPs were synthesized in an aqueous solution using silver nitrate, polyethylene glycol (PEG), and β-D-glucose as a silver precursor, stabilizer, and reducing agent, respectively. The properties of synthesized colloidal Ag NPs were studied at different reaction times. The ultraviolet-visible spectra were in excellent agreement with the obtained nanostructure studies performed by transmission electron microscopy (TEM) and their size distributions. The Ag NPs were characterized by utilizing X-ray diffraction (XRD), zeta potential measurements and Fourier transform infrared (FT-IR). The use of green chemistry reagents, such as glucose, provides green and economic features to this work.

## 1. Introduction

Nanotechnology and especially nanomaterials have received much consideration because their structure and properties differ appreciably from those of molecules, atoms, and bulk materials [1]. The synthesis of metal nanoparticles has been widely discussed in the literature due to their distinctive chemical and physical properties, which have many potential purposes [2,3]. The utilization of non-toxic solvents, biodegradable materials and low-cost green chemicals are central to resources synthesis and processing, considering the green reaction method of these strategies. The stabilizer, reaction medium, and green reducing agent are three key factors in the synthesis and stabilization of metallic nanoparticles [4].

Due to its properties and areas of use, Ag is one of the most studied metals. Stability, morphology, particle size distribution and surface state charge/modification, all play a very significant role and there is much interest in the controlled synthesis of Ag NPs [5,6]. The literature describing the preparation of Ag NPs is particularly broad since the classical colloid methods are combined with modern nanotechnology leading to many procedures for surface modification, particle size control, particle preparation [7,8]. Many synthesis methods have been applied to prepare Ag NPs; chemical reduction in aqueous and non-aqueous media, in soft matrices, and in solid matrices (e.g., mesoporous silicate) [9–11], by applying physical processes/various types of irradiation [12–14], and electrochemical processes [15], in emulsion systems [16]. The Ag NPs are widely used as photo-catalysts [17], catalysts [18], antibacterial [19], biosensor [20], bioimaging [21] and in surface-enhanced Raman spectroscopy [22] as well as chemical analysis [23].

The surface passivation reagents, including surfactant molecules and polymers, are needed to prevent the nanoparticles from aggregation. Polyethylene glycol has been widely applied as an effective passivation agent in the fabrication of Ag NPs and other metal nanoparticles [24]. Recent reports revealed that PEG 200 was effective for the control of size and shape of Ag NPs. The surface modification of these colloidal nanoparticles is very important to facilitate their application to biotechnology and nanocomposites [25].

The synthesis method presented could be useful in providing an economic method for the preparation of compatible, and stable colloidal Ag NPs. After 6 h from reaction times, small colloidal nanoparticles with less than 12 nm in average particle diameter could be obtained. Other benefits of this method contain the physical conditions of the synthesis, such as the use of atmospheric pressure, the lack of need for an additional flow of inert gas and the moderate reaction temperature.

## 2. Results and Discussion

**Figure 1:**
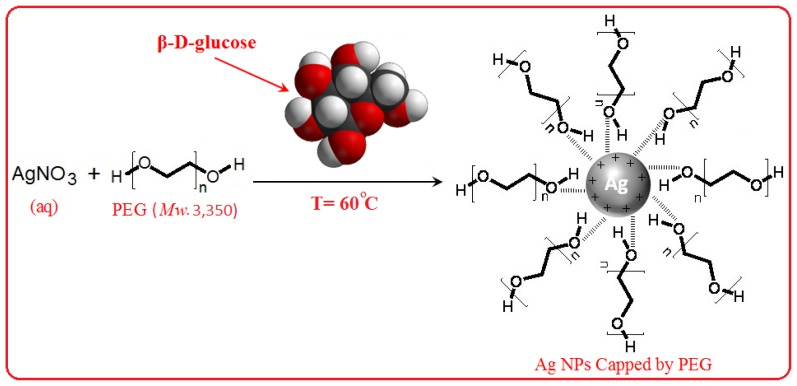
Schematic illustration showing the interactions between the hydroxyl groups in polyethylene glycol (PEG) present with the surface of positive charge of silver nanoparticles [Ag(PEG)].

In this research, PEG was appropriate as a stabilizer and polymeric media for reducing the AgNO_3_ using β-D-glucose as a green reducing agent. The schematic illustration of the synthesis of Ag NPs capped with PEG is depicted schematically in Figure 1.

As shown hydroxyl group of PEG as a capping agent can covered in the surface of Ag NPs. This is due to the surface of Ag NPs which is positively charged. Certainly, we suppose that colloidal stabilization for [Ag(PEG)] occur due to the presence of van der waals forces between the oxygen negatively charged groups present in the molecular structure of the PEG, and the positively charged groups that surround the surface of inert Ag NPs [26]. Figure 1 illustrates the nature of the interaction between the charged Ag NPs and PEG [9,27].

Glucose as an aldehyde can reduce silver ions to Ag NPs and through this process gluconic acid oxidizes itself. The possible chemical equations for preparing the Ag NPs are:

(1) $${{\text{Ag}}^{+}}_{\left(\text{aq}\right)}+{\text{PEG}}_{\left(\text{aq}\right)}\to {{\left[\text{Ag}(\text{PEG})\right]}^{+}}_{\left(\text{aq}\right)}$$

(2) $$2{{\left[\text{Ag}(\text{PEG})\right]}^{+}}_{\left(\text{aq}\right)}+{\text{CH}}_{2}\text{OH}{\left(\text{CHOH}\right)}_{4}\text{CHO}\to 2[\text{Ag}(\text{PEG})]↓+{\text{CH}}_{2}\text{OH}{\left(\text{CHOH}\right)}_{4}\text{COOH}$$

After dispersion of silver ions in the PEG aqueous solution matrix (Equation (1)), PEG reacted with the Ag to form a PEG complex [Ag(PEG)]^+^, which reacted with β-D-glucose to form [Ag(PEG)] due to the reduction of silver ions through the oxidation of glucose to gluconic acid (Equation (2)).

The colorless solution turned to the light yellow, after 1 and 3 h, indicating the initial formation of Ag NPs. When we increased time of the reaction, the color changed to yellow after 6 h, while with continuous stirring at a moderate temperature for 24 and 48 h, the color of the reaction changed to gray and black gray. These observations show that with an increase in reaction time, particle size and aggregation of silver nanocrystal gradually increased together.

### 2.1. UV-Visible Spectroscopy

**Figure 2:**
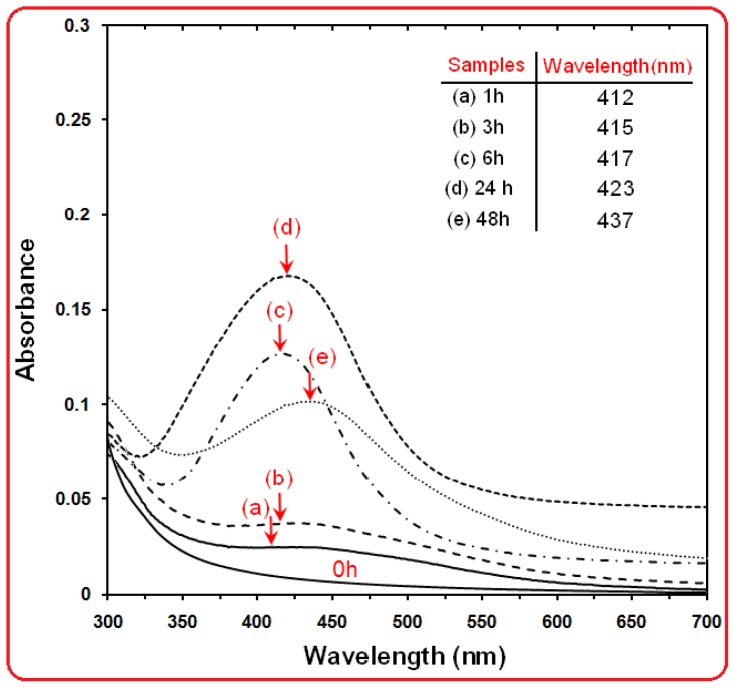
The Ultraviolet visible spectra of Ag NPs prepared in PEG solution at different reaction time in the moderate temperatures.

The formation of Ag NPs in the polymeric media was further determined by using the UV-visible spectroscopy, which was shown on the surface plasmon resonance (SPR) bands. Figure 2 shows that Ag NPs started to form when [Ag(PEG)]^+^ was allowed into the reaction at a moderate temperature as there was no peak at 0 h and the absorbance peak could be seen at different stirring times after the reaction started. Generally, the SPR bands are influenced by the size, shape, morphology, composition and dielectric environment of the prepared nanoparticles [28,29]. Previous studies have shown that the spherical Ag NPs contribute to the absorption bands at around 400 nm in the UV-visible spectra [30]. From this research, the SPR band characteristics of Ag NPs were detected around 412–437 nm (Figure 2a–e), which strongly suggests that the Ag NPs were spherical; this can be confirmed by the TEM results. From Figure 2, as the stirring time of reaction increased, the intensity of the SPR peak also showed a gradual increment until 24 h but after 48 h the SPR peak changed to a broad shape and intensity decreased,. This phenomenon is related to the increased size and also agglomeration of silver nano-crystals [31]. Therefore, this shows that the reduction of the silver ions to silver atoms was continued and resulted in an increase in the concentration of Ag NPs [32].

Thus, there is a normal case in this situation for the SPR absorption band for the particles, which agreed with the TEM results, whereby red-shifts were observed as size increased during the reaction after 1, 3, 6, 24 and 48 h respectively. This can be explained by the multi-layer Mie theory model, which theorizes that the chemical interaction caused the lowered electron conductivity in the outermost atomic layer, and consequently caused the red-shifts [33]. As seen from the Figure 2, it can be observed that 24 h had large absorbance compared to 48 h because the particle size of Ag NPs after 48 h were larger than those at 24 h. Also, absorption spectra of larger metal colloidal dispersions can exhibit broad peaks or additional bands with the lower absorbance in the UV-visible range due to the excitation of plasma resonances or higher multipole plasmon excitation [34]. This phenomenon could be due to the fact that, after reaching a certain particle size, the stabilizer was not able to withhold the nanoparticle’s size effectively, which resulted in its very large size.

### 2.2. Morphologie Study

**Figure 3:**
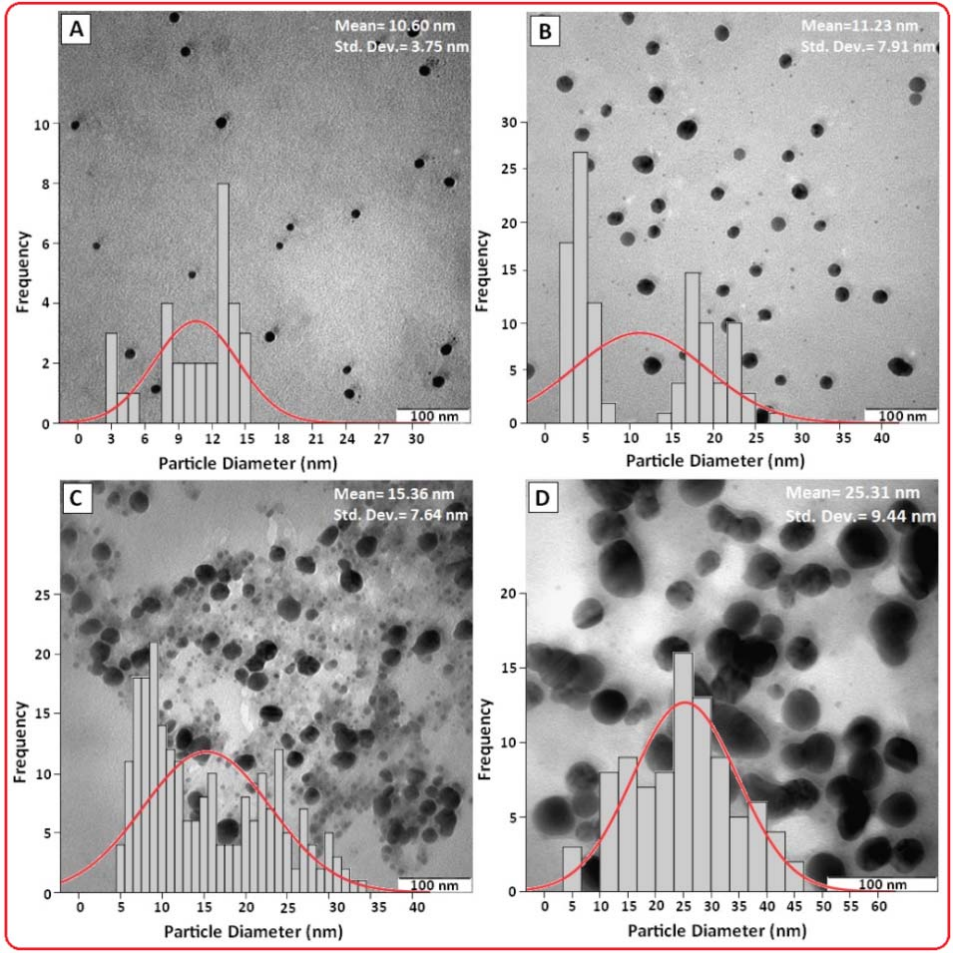
Transmission electron microscopy image and the particle size distribution for Ag NPs in PEG for the stirring times of 3, 6, 24 and 48 h, respectively (**A**–**D**).

Hence, the increase of Ag NPs size from 3 h (10.60 nm) to 6 h (11.23 nm) was very small compared to the increase from 24 h (15.36 nm) to 48 h (25.31 nm) as demonstrated by the TEM. The TEM images and their corresponding particle size distributions of Ag NPs at different periods of time are shown in Figure 3. The TEM images and their size distributions revealed that the mean diameters and standard deviation of Ag NPs were about 10.60 ± 3.75, 11.23 ± 7.91, 15.30 ± 7.64 and 25.31 ± 9.44 nm for 3, 6, 24 and 48 h (Figure 3A–D), respectively. The total numbers of Ag NPs counted for each TEM images were about 32, 107, 226 and 64 for 3, 6, 24 and 48 h respectively. These results confirmed that, with increasing reaction time, mean diameters and standard deviations of the Ag NPs gradually increases.

### 2.3. Powder X-ray Diffraction

**Figure 4:**
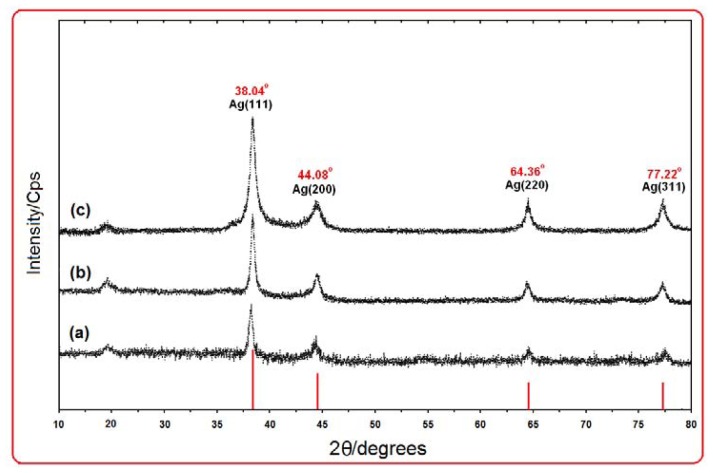
X-ray diffraction patterns of Ag NPs synthesized in PEG after 6, 24 and 48 h respectively (**a**–**c**).

Figure 4 shows the XRD patterns of Ag NPs formed in the 6 h, 24 h, and 48 h from stirring time of reaction, which indicates the formation of the silver crystalline structure. The XRD peaks in the wide angle range of 2θ (10° < 2θ < 80°) ascertained that the peaks in 38.04°, 44.08°, 64.36° and 77.22° can be attributed to the 111, 200, 220, and 311 crystalline structures of the face centered cubic (fcc) synthesized silver nano-crystal, respectively (Ag XRD Ref. No. 00-087-0719) [19]. The intensities of 111, 200, 220 and 311 reflections due to the Ag NPs phase were also found to increase along with the increased Ag NPs in the polymeric media (Figure 4a–c). The peaks showed that the main composition of nanoparticles was silver and no obvious other peaks present as impurities were found in the XRD patterns. Therefore, this gives clear evidence for the presence of Ag NPs in the [Ag (PEG)]. The average particle size of silver nanoparticles can be calculated using Debye-Scherrer equation:

(3) $$n=\frac{K\lambda }{\beta \;\text{cos }\theta }$$

Where *K* is the *Scherrer* constant with value from 0.9 to 1 (shape factor), where *λ* is the X-ray wavelength (1.5418 Å), *β*_1/2_ is the width of the XRD peak at half height and *θ* is the Bragg angle. From the Scherrer equation, the average crystallite size of silver nanoparticles for 6, 24 and 48 h from the time of reaction are found to be around 10–25 nm, which were also in line with the observation of the TEM results discussed later. A small peak observed at around 2θ value of 19° for all samples is due to organic moiety (PEG).

### 2.4. Surface Chemistry (FT-IR)

**Figure 5:**
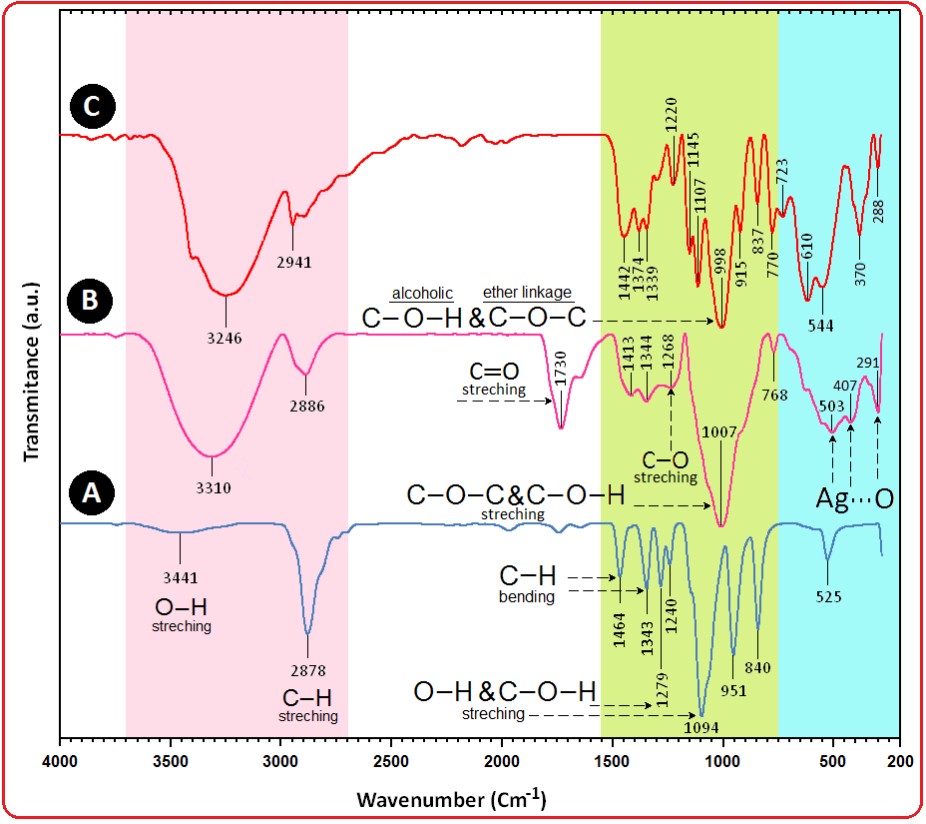
Fourier transform infrared spectra for PEG (**A**), [Ag(PEG)] for the stirring time of 48 h (**B**) and β-D-glucose (**C**).

On the other hand, as for the β-D-glucose spectrum (Figure 5C), the absorption bands at 3246 cm^−1^ was due to the O–H stretching band, 2901 cm^−1^ was due to the aliphatic C–H stretching, 1442, 1374 and 1339 cm^−1^ were due to C-H bending vibrations, and also the combination band of O–C–H and C–O–H deformation is calculated from 1442 to 1339 cm^−1^. Then the in plane C–H and O–H deformation from 1220 to 998 cm^−1^ can be observed. In the region from 1145 to 554 cm^−1^, the C–O and C–C groups’ vibration modes are present and the carbohydrates generally show their characteristic bands [35].

The interaction of Ag NPs obtained with PEG and gluconic acid products by reduction of β-D-glucose compound were confirmed by FT-IR spectra (Figure 5B). Intense absorptions are observed at 1730, 1630 and 1007 cm^−1^. The IR band at 1730 cm^−1^ is characteristic of the C=O stretching mode of the carboxylic acid group for gluconic acid. The bands due to C–O stretching mode were merged in the very broad envelope centered on 1268 and 1007 cm^−1^ arising from C–O, C–O–C stretches and C–O–H bends vibrations of Ag NPs in PEG. Also, the aliphatic C–H stretching, in 1413 and 1344 cm^−1^ were due to C–H bending vibrations [36]. After the bio-reaction of β-D-glucose with the AgNO_3_ in the PEG matrix, the created peak in 1730 cm^−1^ certified to the binding of –C=O for carboxylic acid in gluconic acid, and the shift in the peak at 1007 cm^−1^ towards lower frequency compared to peak in 1094 cm^−1^ for PEG is attributed to the binding of C–C–O and C–C–H groups with nanoparticles are shown in Figure 5B [37,38].

The broad peaks in 503, 407 and 291 cm^−1^ related to Ag NPs banding with oxygen from hydroxyl groups of PEG chains. Therefore, the FT-IR spectra showed the existence of van der Waals interactions between the chain of PEG and Ag NPs in the polymeric media [39].

### 2.5. Zeta Potential Measurement

**Figure 6:**
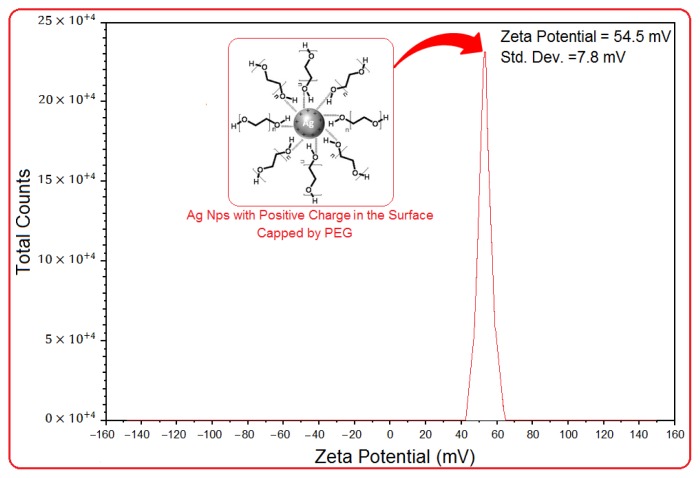
Zeta potential measurements for [Ag(PEG)] NPs after 6 h of stirring time.

As shown in Figure 6, the Ag NPs obtained possess a positive zeta potential value. Zeta potential is an essential parameter for the characterization of stability in aqueous nanosuspensions. A minimum of ±30 mV zeta potential values is required for indication of stable nanosuspension [40]. At 6 h of stirring time, the zeta potential was equal to 54.5 ± 7.8 mV. So, this result clearly indicated that the particles are fairly stable due to the electrostatic repulsion.

## 3. Experimental Section

### 3.1. Materials and Method

All reagents in this effort were analytical grade and were used as received without further purification. AgNO_3_ (99.98%) was used as a silver precursor, and was provided by Merck, Germany. PEG (Mw 3350) used as a stabilizer for the preparation of Ag NPs which was purchased from Sigma-Aldrich (USA). Meanwhile, the β-D-glucose was used as a green reducing of silver ions to Ag atoms was obtained from BDH Chemical Ltd., Poole, UK. All solutions were freshly prepared using double distilled water and kept in the dark to avoid any photochemical reactions. All glassware used in experimental procedures were cleaned in a fresh solution of HNO_3_/HCl (3:1, v/v), washed thoroughly with double distilled water, and dried before use.

### 3.2. Synthesis of Ag NPs by Using Green Method

The preparation of Ag NPs in the PEG matrix is quite directly forward. In a typical synthesis, 10 mL of a 1.0 M solution of AgNO_3_ was added to 200 mL of a 0.1 wt% aqueous solution of soluble PEG to obtain the clear solution [Ag(PEG)]^+^_(aq)_. After complete dissolution of these components, 20 mL of a 1.0 M aqueous solution of β-D-glucose was added and further stirred. The solution obtained was distributed into 5 cuvettes, and the prepared solutions were stirred and maintained for different periods of time: 1 (a), 3 (b), 6 (c), 24 (d), and 48 h (e), respectively. Throughout the reduction process, all solutions were kept at a temperature of 45 °C in the dark place to avoid any photochemical reactions. The mixture was heated to 45 °C and was maintained at this temperature for 1, 3, 6, 24 and 48 h (a–e) respectively. The obtained colloidal suspension of [Ag(PEG)] was then centrifuged at 20,000 rpm for 15 min, these precipitates were washed four times using double distilled water in order to remove the silver ion residue and dried overnight at 40 °C under a vacuum.

### 3.3. Characterization Methods and Instruments

The prepared Ag NPs were characterized using X-ray diffraction (XRD), transmission electron microscopy (TEM), ultraviolet-visible spectroscopy, Fourier transform infrared (FT-IR) spectroscopy and zeta potential measurements. The XRD patterns were recorded at a scan speed of 2° min^−1^. Meanwhile, the structures of the produced Ag NPs were examined using Shimadzu PXRD-6000, powder x-ray diffraction. Moreover, TEM observations were carried out using the Hitachi H-7100 electron microscopy, whereas the particle size distributions were determined using the UTHSCSA Image Tool software (Version 3.00). To ensure the formation of Ag NPs, the colloids solutions were tested for their optical absorption property using a Shimadzu H.UV, 1650 PC UV-visible spectrophotometer over the range of 300 to 700 nm. Meanwhile, the FT-IR spectra were recorded over the range of 200–4000 cm^−1^ utilising the Series 100 Perkin Elmer FT-IR 1650 spectrophotometer. The zeta potential measurements were also performed using a Zetasizer Nano-ZS (Malvern Instruments, Worcestershire, UK).

## 4. Conclusions

In summary, we have described a simple and green method to synthesize colloidal Ag NPs by using green reducing agents which requires no special physical conditions. Ag NPs were successfully synthesized under moderate temperature (45 °C) at different stirring times of reaction. The formation of Ag NPs was confirmed in the UV-visible absorption spectra, which showed the SPR band characteristics of Ag NPs in the range of 412–437 nm. The XRD results confirmed that the Ag NPs possessed a face-centered cubic crystal structure. In addition, this also revealed that Ag NPs were the main composition present in the nanocomposites without any contamination peaks. The TEM images showed that the Ag NPs were in spherical shape and the average diameters of the particles were 10.60, 11.23, 15.30 and 25.31 nm for the stirring times of 3, 6, 24 and 48 h, respectively. The FTIR spectrum suggested the complexation present between PEG and Ag NPs, enabling the formation of metallopolymer Ag[PEG] and the stability of the Ag NPs was confirmed with the zeta potential measurements.
